# Laminin fragments conjugated with perlecan’s growth factor-binding domain differentiate human induced pluripotent stem cells into skin-derived precursor cells

**DOI:** 10.1038/s41598-023-41701-5

**Published:** 2023-09-04

**Authors:** Yoriko Sugiyama-Nakagiri, Shiho Yamashita, Yukimasa Taniguchi, Chisei Shimono, Kiyotoshi Sekiguchi

**Affiliations:** 1 Kao Corporation, 2602, Akabane Ichikai-Machi, Haga-gun, Tochigi 321-3497 Japan; 2 Division of Matrixome Research and Application, Institute for Protein Research, Osaka University, Suita, Osaka Japan

**Keywords:** Biological techniques, Stem cells

## Abstract

Deriving stem cells to regenerate full-thickness human skin is important for treating skin disorders without invasive surgical procedures. Our previous protocol to differentiate human induced pluripotent stem cells (iPSCs) into skin-derived precursor cells (SKPs) as a source of dermal stem cells employs mouse fibroblasts as feeder cells and is therefore unsuitable for clinical use. Herein, we report a feeder-free method for differentiating iPSCs into SKPs by customising culture substrates. We immunohistochemically screened for laminins expressed in dermal papillae (DP) and explored the conditions for inducing the differentiation of iPSCs into SKPs on recombinant laminin E8 (LM-E8) fragments with or without conjugation to domain I of perlecan (PDI), which binds to growth factors through heparan sulphate chains. Several LM-E8 fragments, including those of LM111, 121, 332, 421, 511, and 521, supported iPSC differentiation into SKPs without PDI conjugation. However, the SKP yield was significantly enhanced on PDI-conjugated LM-E8 fragments. SKPs induced on PDI-conjugated LM111-E8 fragments retained the gene expression patterns characteristic of SKPs, as well as the ability to differentiate into adipocytes, osteocytes, and Schwann cells. Thus, PDI-conjugated LM-E8 fragments are promising agents for inducing iPSC differentiation into SKPs in clinical settings.

## Introduction

The skin and skin appendages are tissues of the body’s outermost layer and play an important role in bodily protection and as a part of social identity. Technology to regenerate skin tissues can therefore contribute to maintaining homeostasis throughout the body and improving people’s quality of life from a social perspective. It is important to obtain tissue stem cells for skin regeneration, but invasive surgical procedures are required to obtain skin-derived stem cells. In addition, stem cells so isolated may have limited or variable abilities to proliferate and/or differentiate depending on donor age and culture conditions. To circumvent these problems, we developed a method to differentiate human induced pluripotent stem cells (iPSCs) into skin-derived precursor cells (SKPs), the dermal stem cells with self-renewal and multipotent differentiation abilities^1^. However, this method uses mouse fibroblasts as feeder cells and is therefore not suitable for producing iPSC-derived SKPs (iPSC-SKPs) for clinical use. A feeder-free method using xeno-free scaffold materials therefore needs to be developed for clinical application of iPSC-SKPs.

In vivo, cells interact with their surrounding extracellular matrix (ECM) and control their activities such as proliferation, differentiation, and cell death through the signals transduced by the integrin family of adhesion receptors in conjunction with the signals elicited by a variety of growth factor receptors^2^. Thus, the control of fate and function of stem cells by manipulating the molecular composition and stiffness of ECM is an attractive approach. For example, mesenchymal stem cells differentiate into nerves, skeletal muscles, and osteoblasts depending on scaffold stiffness^3^. Laminin (LM) 511, the major component of the basement membrane (BM) underlying multipotent epiblasts^4,5^, is suitable for maintaining undifferentiated human embryonic stem cells and iPSCs^6,7^. COL17A1 expressed in keratinocytes contributes to the maintenance of melanocyte stem cells of hair follicles^8^. Thus, ECM proteins are promising agents to govern the nature of stem cells and their niches.

LMs are heterotrimeric proteins of α, β, and γ chains that are associated at a ratio of 1:1:1. There are five α chains (α1, α2, α3, α4, and α5), three β chains (β1, β2, and β3), and three γ chains (γ1, γ2, and γ3), yielding 16 LM isoforms with distinct chain combinations^9^. Among these LM isoforms, full-length LM511 and its functionally active fragment (LM511-E8 fragment) have been produced recombinantly and used as culture substrates for maintaining undifferentiated iPSCs^10^. Furthermore, the efficiency of directed differentiation of human iPSCs can be enhanced by customising the LM-E8 fragments used as culture substrates: LM411, the LM isoform expressed in blood vessels, and its E8 fragment promote the directed differentiation of iPSCs into blood endothelial cells^11^. A mixture of LM511-E8 and LM111-E8 fragments increases the induction efficiency of hepatocyte progenitor cells from iPSCs^12^. The LM332-E8 fragment preferentially supports the differentiation of corneal epithelial cells from iPSCs^13^. The LM511-E8 fragment supports the induction of neural differentiation of iPSCs, thereby enabling the clinical application of iPSC-derived dopaminergic progenitors for cell replacement therapy of Parkinson’s disease^14^.

Directed differentiation of pluripotent stem cells into distinct cell lineages is driven by ECM and a combination of growth factors, many of which bind to heparin/heparan sulphate chains. Perlecan, a major heparan sulphate proteoglycan of BMs, captures a variety of growth factors including fibroblast growth factors, vascular endothelial growth factors, platelet-derived growth factors, activins, and bone morphogenetic proteins through its domain I (PDI) that harbours heparan sulphate chains, thereby regulates their activities as well as tissue distributions^15,16^. It is therefore important to incorporate the growth factor-modulating function of heparan sulphate proteoglycans such as perlecan into the culture substrates for directed differentiation of iPSCs.

In this study, we focused on the LM isoforms expressed in the dermal papillae (DP) of hair follicles, where SKPs are localised in vivo^17^, and investigated the culture substrates suitable for the directed differentiation of SKPs from iPSCs. Our results show that the E8 fragments of the LM isoforms expressed in DP, including LM111-E8, LM121-E8, LM332-E8, LM421-E8, LM511-E8, and LM521-E8, are effective substrates for inducing the differentiation of iPSC-SKPs. We also utilised chimeric LM-E8 fragments conjugated with the growth factor-binding PDI domain of perlecan and found that the chimeric LM-E8 fragments substantially improves the efficiency of directed differentiation of iPSCs into SKPs. These findings offer culture substrates suitable for the directed differentiation of iPSCs to SKPs in clinical settings.

## Results

### Identification of LM chains expressed in human dermal papillae

**Figure 1:**
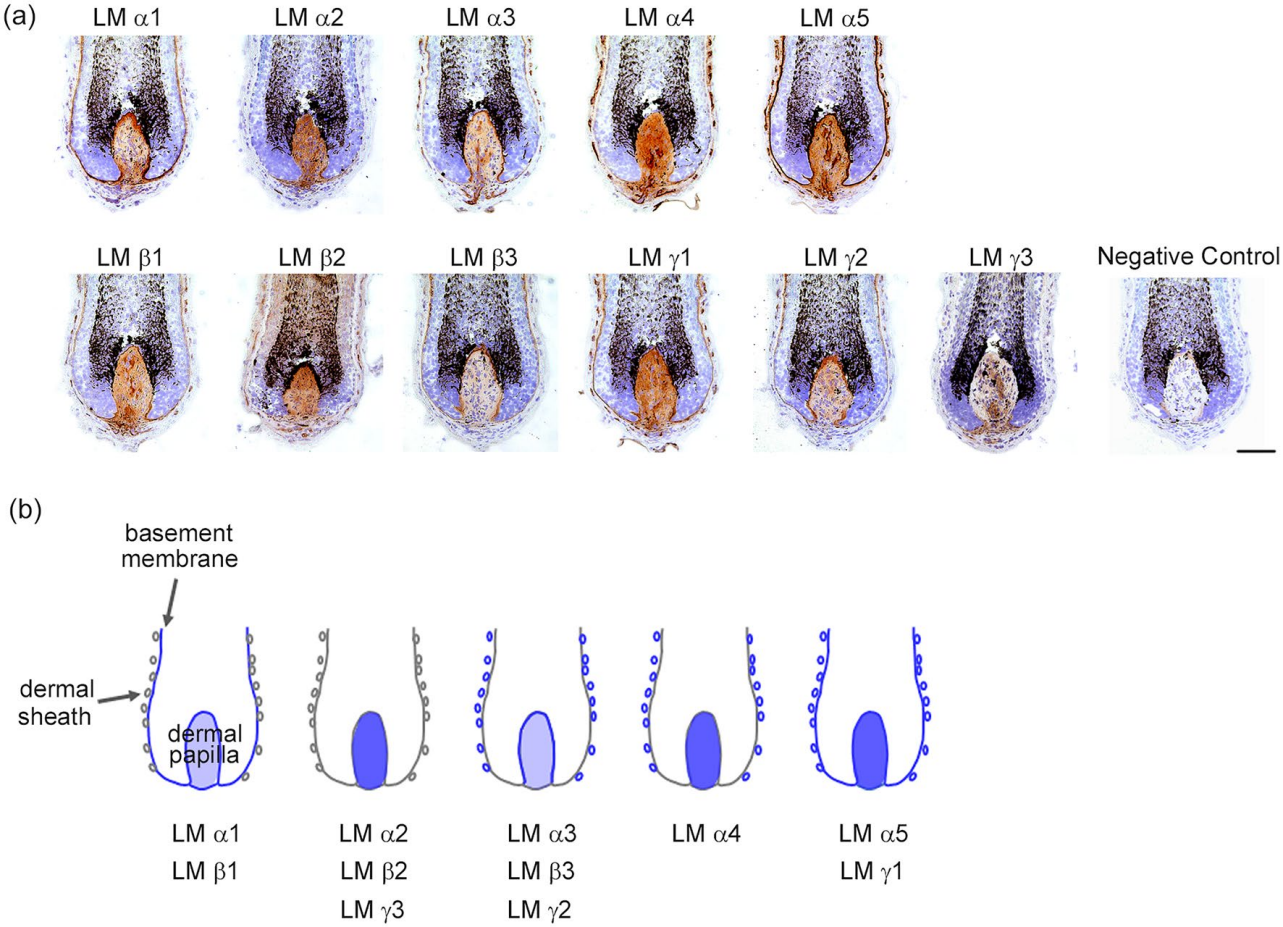
LM chain expression in human dermal papillae. (**a**) Identification of LM chains expressed in human dermal papillae (DP) using immunohistochemistry. All LM chains were expressed in DP. Negative control is shown in the bottom right. Scale bar = 100 μm. (**b**) Schematic diagram of the results of LM chain expression in human DP.

To identify LM isoforms suitable for differentiating iPSC-SKPs, we first analysed the LM chains expressed in human hair follicle DP using immunohistochemistry. All LM chains were detected in DP with distinct localisation patterns (Fig. 1a). LM α1 and β1 were detected in the epithelial BM of hair follicles and within the entire DP, whereas LM α2, β2, and γ3 were selectively expressed in DP. LM α3, β3, and γ2 were expressed in the BMs in contact with DP and dermal sheath cells. LM α4 was localised in DP and dermal sheath cells, and LM α5 and γ1 were in the epithelial BM, DP, and dermal sheath cells. A schematic of LM expression in the human hair follicle is depicted in Fig. 1b.

### Generation of iPSC-SKPs using recombinant LM-E8 fragments

**Figure 2:**
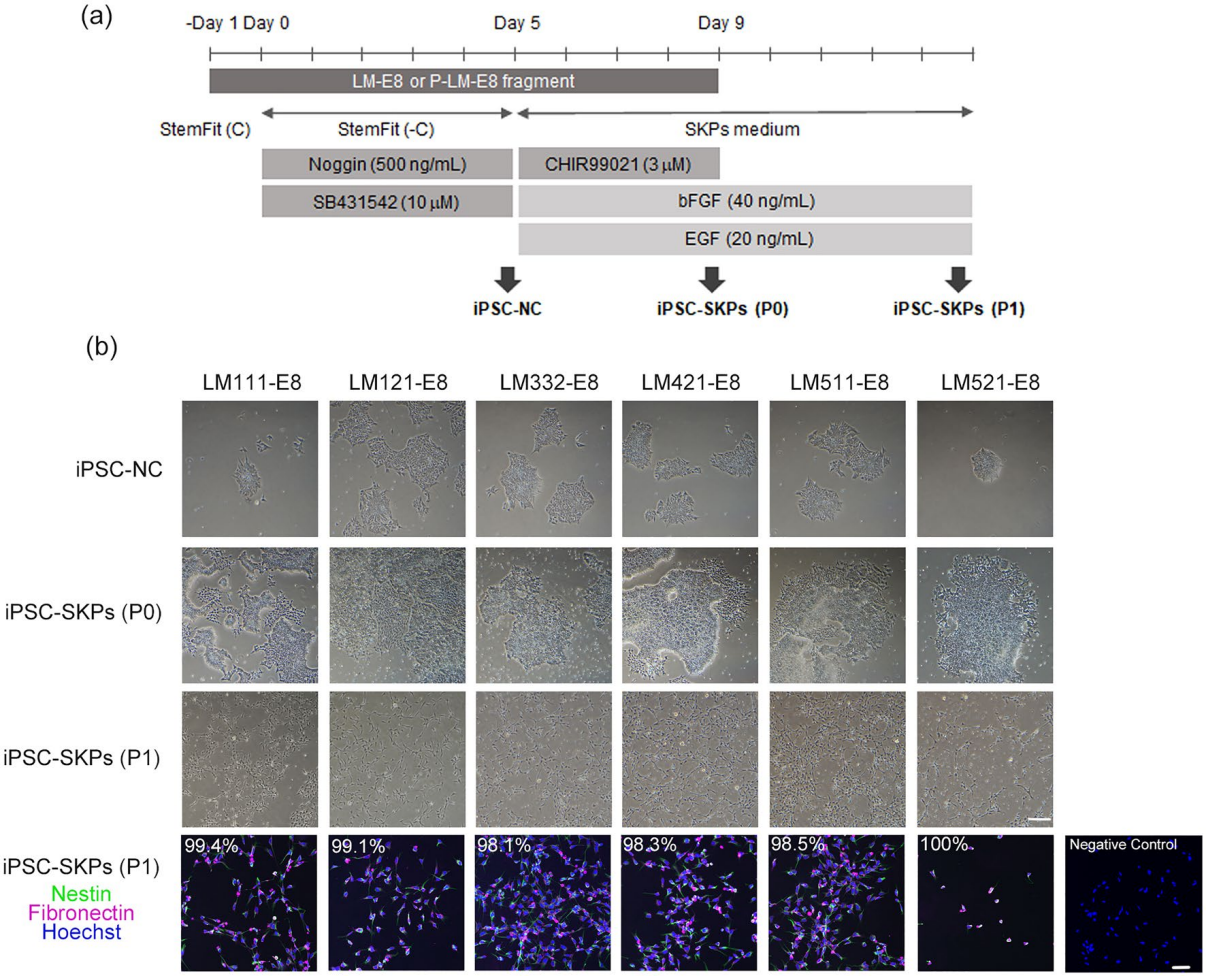
Generation of SKPs from human iPSCs on LM-E8 fragments. (**a**) Schematics of the protocol used to differentiate iPSC-SKPs on LM-E8 fragments. Before the initiation of SKP differentiation, undifferentiated iPSCs were precultured on LM-E8 fragments for 7 days. When iPSC colonies reached 80–90% confluence, the cells were detached and seeded in StemFit medium containing 10 µM Y-27632 (− Day1) on each LM-E8 fragment. After 24 h (Day 0), the medium was switched to StemFit medium without liquid C, containing 500 ng/mL noggin and 10 µM SB431542 for neural crest differentiation. After 5 days of culture, the StemFit medium was removed, and the cells were cultured for 4 days in SKP medium containing DMEM/F12, 2% B27 supplement, 40 ng/mL bFGF, 20 ng/mL EGF, and 3 µM CHIR99021 for SKP differentiation. After 4 days of culture, the cells were dissociated using Accutase and subcultured on new uncoated dishes in SKP medium without CHIR99021. (**b**) Differentiation of iPSC-SKPs on various LM-E8 fragments. iPSCs (1231A3) were induced to differentiate to SKPs as schematically shown in (**a**). Phase contrast images of iPSC-SKPs at different stages of differentiation, that is, iPSC-NC (Day 5; iPSC-derived neural crest cell), iPSC-SKPs (P0) (Day 9; iPSC-derived SKPs, passage 0), and iPSC-SKPs (P1) (Day 14; iPSC-derived SKPs, passage 1), are shown. The cells immunostained for nestin (green) and fibronectin (magenta) on Day 14 are shown in the bottom row with the percentages of nestin-/fibronectin-positive cells. Negative control is shown in the bottom right. Scale bars, 200 μm for phase-contract images and 50 μm for immunofluorescence images.

To identify LM isoforms suitable for the induction of SKPs from iPSCs, we induced the differentiation of iPSCs to SKPs on various recombinant LM-E8 fragments, based on the protocol established using mouse fibroblasts as feeder cells^1^ (Fig. 2a). Before the initiation of SKP differentiation, undifferentiated iPSCs were precultured on the same LM-E8 fragment. When iPSC colonies reached 80–90% confluence, the cells were detached and seeded on each LM-E8 fragment (− Day1). After 24 h of culture, the medium was switched to the medium containing noggin and SB431542 for neural crest differentiation. After 5 days of culture, the medium was switched to Dulbecco’s modified Eagle’s medium (DMEM)/F12 medium containing basic fibroblast growth factor (bFGF), epidermal growth factor (EGF), and CHIR99021 (‘SKP medium’), and the cells were induced to differentiate into SKPs. After 4 days of culture, the cells were dissociated and subcultured on new uncoated dishes in SKP medium without CHIR99021.

Undifferentiated iPSCs did not proliferate on LM211-E8 or LM411-E8, although temporary adhesion was observed after seeding (data not shown). In contrast, undifferentiated iPSCs stably adhered and differentiated into iPSC-SKPs on LM111-E8, LM121-E8, LM332-E8, LM421-E8, LM511-E8, and LM521-E8 fragments (Fig. 2b). In addition, we examined the expression of nestin and fibronectin, which are well-characterised markers for SKPs^18^, in the resulting cells using immunofluorescence staining. More than 98% of the cells differentiated on each LM-E8 fragment were positive for both nestin and fibronectin, confirming that the cells induced were iPSC-SKPs (Fig. 2b and Supplementary Fig. S1). Although the induction efficiency on LM-E8 fragments varied between experiments and between LM-E8 fragments used, it was lower than that achieved by the original protocol using mouse feeder cells (Supplementary Fig. S2).

### Differentiation of iPSC-SKPs using LM-E8 fragments conjugated with the growth factor-binding domain of perlecan

**Figure 3:**
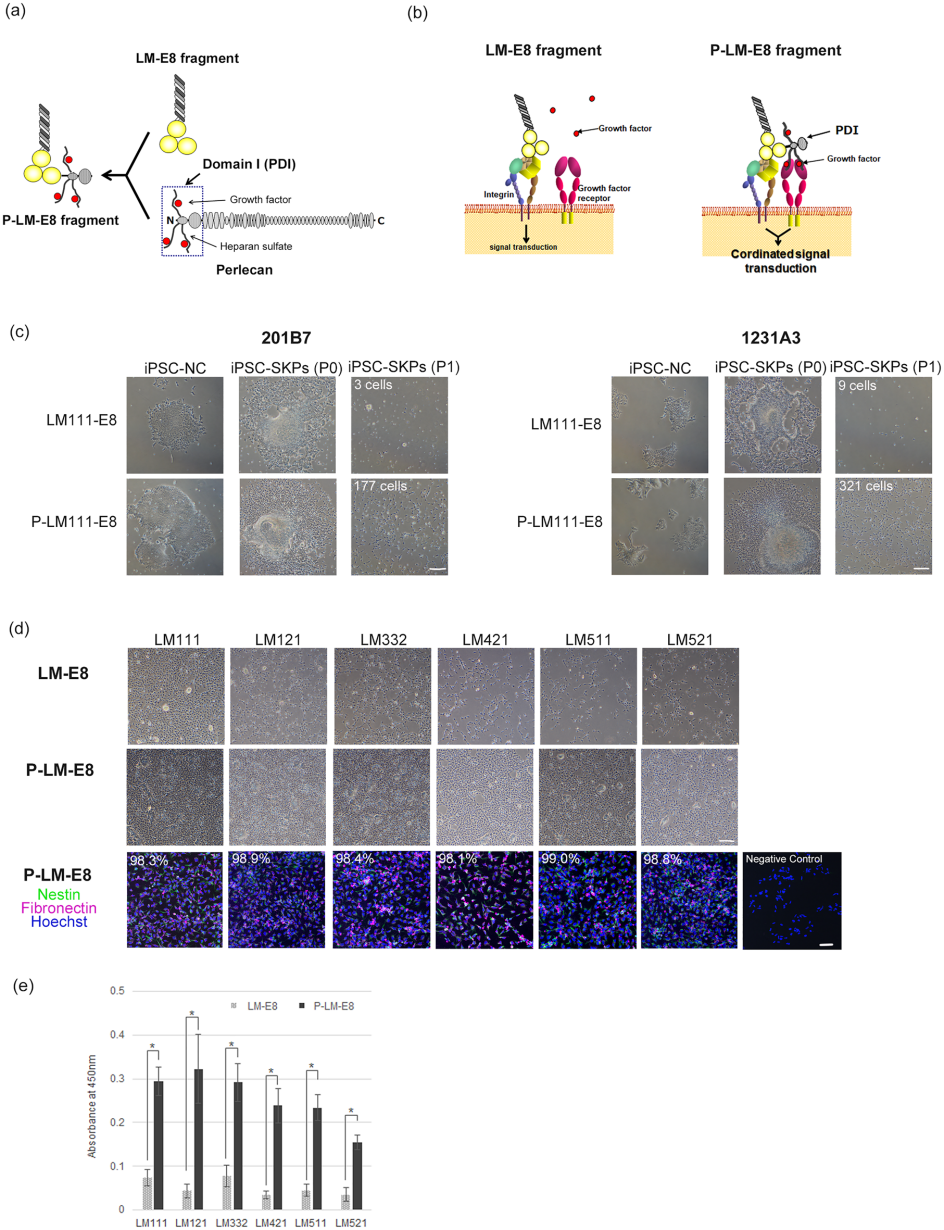
Improved iPSC-SKP differentiation on P-LM-E8 fragments. (**a**) Structure of P-LM-E8 fragment. The heparan sulphate chain-attached domain I of perlecan (PDI) was fused to the C-terminus of the LM α chain. (**b**) Signal transduction on LM-E8 (left) and P-LM-E8 (right) fragments. On the LM-E8 fragment, the signals from LM and those from growth factors are independently transduced via integrin and growth factor receptor. On P-LM-E8 fragments, the growth factors are captured by heparan sulphate chains attached to perlecan domain I, bringing their receptors close to integrin, thereby integrating the signals downstream of integrin and those downstream of growth factor receptor. (**c**) Differentiation of iPSC-SKPs on the P-LM111-E8 fragment. Before the induction of differentiation, undifferentiated iPSCs were precultured on LM111-E8. The acclimated cells were seeded on a culture dish coated with LM111-E8 or P-LM111-E8 and differentiated into iPSC-SKPs. The differentiated cells on Day 14, that is, iPSC-SKPs (P1), were counted at three non-overlapping fields and expressed as the number of cells per mm^2^ (shown in the upper left corner of each panel). Both human iPSC lines (201B7 and 1231A3) were induced to differentiate into iPSC-SKPs on the P-LM111-E8 fragment more efficiently than on the LM111-E8 fragment, as evidenced by the increased number of differentiated cells. Scale bar, 200 μm. (**d**) Differentiation of iPSC-SKPs on a panel of P-LM-E8 fragments. The phase-contrast images of iPSC-SKPs (P1) differentiated on LM-E8 (upper row) and P-LM-E8 (middle row) fragments are shown along with the immunofluorescence staining of the cells differentiated on P-LM-E8 fragments (bottom row) for nestin and fibronectin. All P-LM-E8 fragments examined induced iPSC-SKPs more efficiently than LM-E8 fragments, as evidenced by the increase in the cell number on Day 14. Most of the resulting cells were positive for nestin and fibronectin. The percentages of the nestin-/fibronectin-positive cells are shown in the upper left corner. Negative control is shown in the bottom right. Scale bars, 200 μm for phase-contract images and 50 μm for immunofluorescence images. (**e**) Differentiation efficiency of iPSC-SKPs on various LM-E8 and P-LM-E8 fragments. The differentiation efficiency was assessed by quantifying the number of live SKPs on Day 14 using the Cell Counting Kit 8 (Abcam) in one representative experiment. Bars, mean ± SD. *p < 0.001.

To improve the induction efficiency of iPSC-SKPs on LM-E8 fragments, we modified LM-E8 fragments by conjugating the growth factor-binding PDI domain of perlecan, a major heparan sulphate proteoglycan of BMs, to the C-termini of LM α chains (Fig. 3a). The resulting PDI-conjugated LM-E8 fragments, referred to as P-LM-E8 fragments, are expected to possess the ability to not only bind to integrins but also capture and prime growth factors, thereby integrating the signals downstream of integrins and growth factor receptors and promoting the differentiation of iPSCs to SKPs (Fig. 3b). To examine the effect of P-LM-E8 fragments on the efficiency of SKP differentiation, we precultured two lines of iPSCs, 201B7 and 1231A3, on LM111-E8 for 7 days, and then the acclimated cells were seeded on culture dishes coated with LM111-E8 or P-LM111-E8 fragment, followed by the induction of differentiation into iPSC-SKPs. Both iPSC lines differentiated into iPSC-SKPs on the P-LM111-E8 fragment more efficiently than on the LM111-E8 fragment, as evidenced by the increase in the number of differentiated cells (Fig. 3c). We also examined iPSC-SKP differentiation on P-LM-E8 fragments of other LM isoforms. As was the case with LM111-E8, undifferentiated iPSCs were precultured on each LM-E8 fragment without PDI before inducing differentiation, seeded on the LM-E8 or P-LM-E8 fragment, and differentiated into iPSC-SKPs. The number of differentiated cells significantly increased on P-LM-E8 fragments compared with that on LM-E8 fragment (Fig. 3d). Furthermore, more than 98% of the differentiated cells on P-LM-E8 fragments were positive for nestin and fibronectin irrespective of the LM isoforms (Fig. 3d and Supplementary Fig. S3), indicating that most, if not all, of the cells differentiated on P-LM-E8 fragments are iPSC-SKPs. Thus, we used the number of iPSC-SKPs (P1) as an index for differentiation induction efficiency without further purification of iPSC-SKPs. The efficiency of SKP differentiation increased by approximately 4–8 times on P-LM-E8 fragments compared with that on LM-E8, irrespective of LM isoforms (Fig. 3e).

### Characterisation of iPSC-SKPs differentiated on P-LM111-E8 fragment

**Figure 4:**
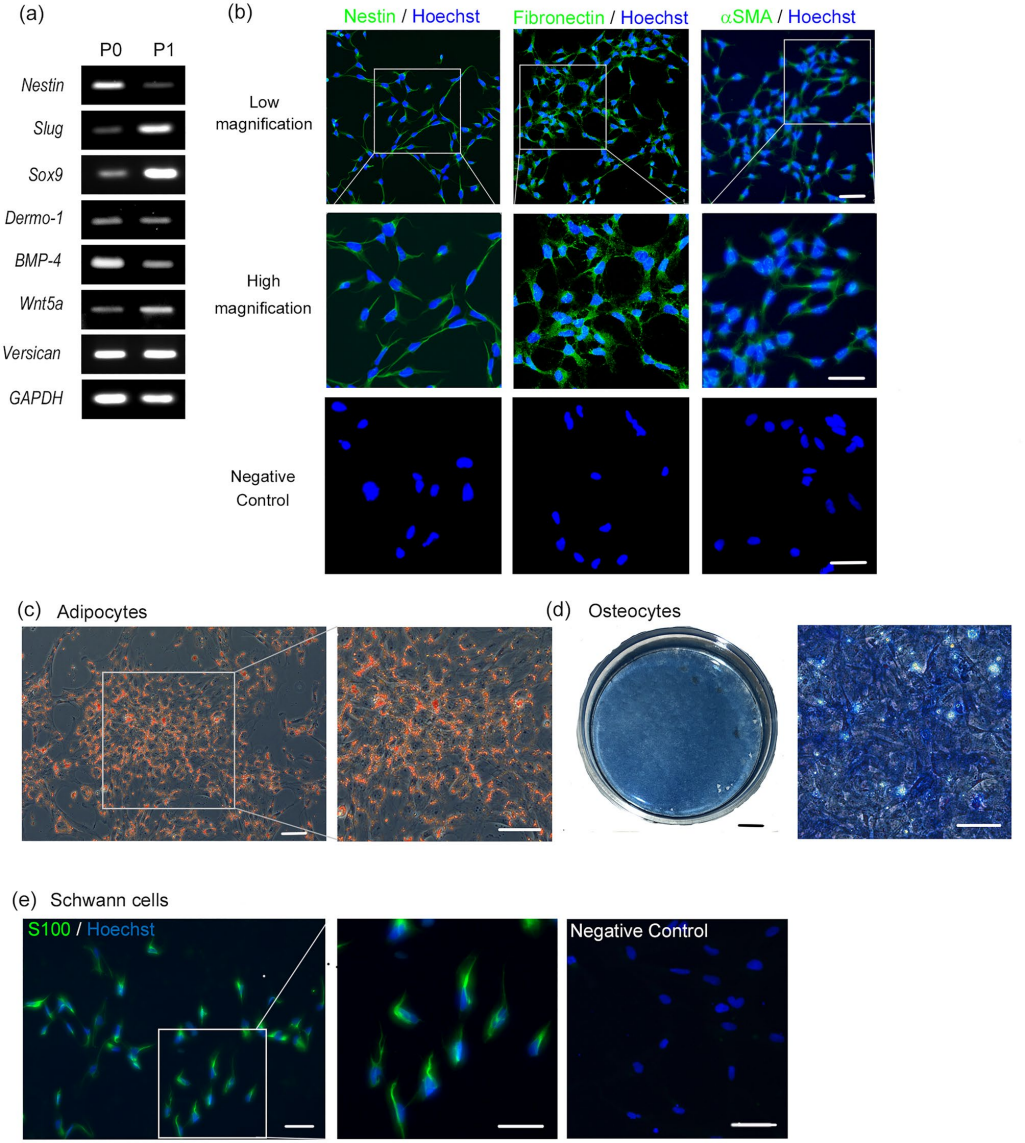
Characterisation of iPSC-SKPs differentiated on P-LM111-E8 fragments. (**a**) RT-PCR analysis of the genes expressed in iPSC-SKPs differentiated on P-LM111-E8 fragment. GAPDH was used as a loading control. (**b**) Immunocytochemical detection of nestin, fibronectin, and αSMA in iPSC-SKPs differentiated on P-LM111-E8 fragment. The boxed areas in the upper panels are enlarged in the middle panels. The bottom panels show negative controls. Scale bars, 100 μm for the upper panels and 50 μm for the middle and lower panels. (**c**–**e**) The ability of iPSC-SKPs induced on P-LM111-E8 fragment to differentiate to adipocytes (**c**), osteocytes (**d**), and Schwann cells (**e**). Adipogenic differentiation of iPSC-SKPs was confirmed by Oil Red O staining (**c**). Osteogenic differentiation was confirmed by ALP staining (**d**). Schwann cell lineages were detected by S100β immunochemical staining (**e**). Negative control is shown in the right. The boxed areas in the left panels in (**c**) and (**e**) are enlarged in the right panels. The overall view of the plate stained for ALP is shown on the left in (**d**). Scale bars, 100 μm for adipocytes (left and right), 5 mm (left) and 50 μm (right) for osteocytes, and 50 μm for Schwann cells.

To characterise iPSC-SKPs differentiated on P-LM-E8 fragments, we examined the expression of genes that have been well-characterised in SKPs^18^. RT-PCR analyses showed that iPSC-SKPs differentiated on P-LM111-E8 fragment expressed RNA transcripts for *Nestin*, *Slug*, *Sox9*, *Dermo-1*, *BMP-4*, *Wnt5a*, and *Versican*, all of which are genes expressed in SKPs isolated from the human dermis^18^ and iPSC-SKPs induced on mouse feeder cells^1^ (Fig. 4a and Supplementary Fig. S4). Immunocytochemistry showed that nestin, fibronectin, and αSMA, known SKP markers, were expressed in iPSC-SKPs differentiated on the P-LM111-E8 fragment (Fig. 4b).

SKPs are defined by their ability to differentiate into adipocytes, osteocytes, and Schwann cells in vitro^17–19^. The iPSC-SKPs differentiated on mouse feeder cells exhibited the same differentiation potential as the SKPs isolated from human dermis^1^. To examine whether the iPSC-SKPs differentiated on P-LM111-E8 fragment were functionally equivalent to the iPSC-SKPs induced on mouse feeder cells, we examined their potential to differentiate into adipocytes, osteocytes, and Schwann cells (Fig. 4c–e). Adipocytes were induced in the medium containing adipogenic factors, that is, 3-isobutyl-1-methylxanthine (IBMX), insulin, and dexamethasone. After 2 weeks of adipogenic differentiation, more than 95% of the resultant cells displayed significant lipid accumulation in the cytoplasm, as demonstrated by staining with Oil Red O (Fig. 4c). Similarly, after 2 weeks of osteogenic differentiation, the resulting cells were positive for the expression of ALP, a marker for osteocytes (Fig. 4d). The entire field of view was ALP-positive at a low magnification, indicating that almost all cells had differentiated into osteocytes. Schwann cell differentiation was induced with the neural cell induction medium containing forskolin and heregulin-1β. The resulting cells expressed S100β, a marker for Schwann cells, after 3 weeks of differentiation (Fig. 4e). Almost all resulting cells were positive for S100β. These results demonstrate that the iPSC-SKPs differentiated on P-LM111-E8 fragments are multipotent and retain the potential to differentiate into adipogenic, osteogenic, and neurogenic lineages, as with the SKPs isolated from human dermis and the iPSC-SKPs induced on mouse feeder cells.

## Discussion

In the present study, we showed that P-LM-E8 fragments conjugated with the growth factor-binding domain of perlecan are useful as culture substrates for differentiating human iPSCs into SKPs. Immunohistochemical analyses showed that all LM chains, including five α chains, three β chains, and three γ chains, are expressed in DP with distinct localisation patterns. The E8 fragments of the LM isoforms expressed in DP support the directed differentiation of iPSCs into SKPs, but P-LM-E8 fragments are more potent than unmodified LM-E8 fragments in inducing iPSC-SKPs. In addition, the cells obtained on P-LM111-E8 fragment are capable of differentiating into adipocytes, osteocytes, and Schwann cells, as has been reported for SKPs isolated from human dermis^17–19^ and iPSC-SKPs induced on mouse feeder cells^1^. These results demonstrate that P-LM-E8 fragments that are modified to capture and activate a variety of growth factors, particularly those capable of binding to heparin/heparan sulphate chains, are useful tools for the targeted differentiation of iPSC-SKPs.

Many cell types, including DP cells, attach to the BM through the interactions of integrins with LMs and receive signals that govern cell fate and behaviour such as multiplication, differentiation, and survival^20^. Induction of directed differentiation of pluripotent stem cells is governed by the signals transduced from integrins, particularly those binding to LMs, and the signals from a defined combination of growth factors. As human stem cells express abundant LM-binding integrins in addition to a variety of growth factor receptors, it is conceivable that the signals downstream of integrins and those of growth factor receptors are introduced cooperatively to ensure their robust fate determination. As recombinantly produced scaffold proteins available to date, such as, full-length LMs and LM-E8 fragments, are only capable of interacting with integrins, the signals elicited by such scaffold proteins exert their function independent of the signals elicited by growth factor receptors (Fig. 3b, left). P-LM-E8 fragments, which comprise the integrin-binding domain of LMs and perlecan’s PDI, which captures a variety of heparin/heparan sulphate-binding growth factors, are expected to bring integrins and growth factor receptors in close proximity and integrate the signals downstream of integrins and those of growth factor receptors, thereby increasing the efficiency of induced differentiation of iPSC-SKPs (Fig. 3b, right). Perlecan is a heparan sulphate proteoglycan ubiquitously present in BMs, including those in contact with DP. It binds to bFGF through its heparan sulphate chains and presents bound bFGF to FGF receptors to potentiate cell proliferation^21^. As the differentiation of iPSCs into SKPs passes through neural crest cells and requires bFGF in the process, the signalling efficiency would be enhanced on P-LM-E8 fragments that capture bFGF on their heparan sulphate chains.

In line with the scheme shown in Fig. 3b, Adachi et al.^22^ reported that P-LM511/521 E8 fragments promote the maturation of hiPSC-derived dopaminergic progenitors in vitro in the presence of brain-derived neurotrophic factor (BDNF) and glial cell line-derived neurotrophic factor (GDNF), as evidenced by the increase in tyrosine hydroxylase activity. When the heparan sulphate chains were removed from P-LM511/521 E8 fragments, the increase in the enzymatic activity was abrogated, underscoring the importance of the heparan sulphate chains attached to the P-LM-E8 fragments. Furthermore, by activating the RAS-ERK signalling pathway, P-LM511/521 E8 fragments promoted maturation and neurite extension of hiPSC-derived dopaminergic progenitors grafted to rodent Parkinson's disease models^22^. These observations, coupled with the results of the present study, underscore the importance of conjugation with the perlecan’s PDI domain to potentiate the capability of LM-E8 fragments as synthetic scaffolds for the manipulation of stem cells both in vitro and in vivo.

Our immunohistochemical staining demonstrated that LM isoforms differ in their localisation patterns within human DP. It is tempting to speculate that the LMs strongly expressed in the BM may contribute to epithelial–mesenchymal interactions, whereas those uniformly expressed in DP may contribute to the interaction between DP cells. Recently, Tsutsui et al.^23^ reported a detailed analysis of the BM composition around the hair follicles in mice. They identified two novel BMs in DP, ‘hook BM’, which protrudes into DP, and ‘mesh BM’, which is detected in a mesh shape within DP, the latter of which is assumed to interact directly with DP cells to control their activation, maintenance of spatial arrangement, and regeneration of hair follicles. Expressed in these BMs are perlecan and LM α2/4/5, β1/2, and γ1/3 chains, all of which were strongly expressed in the BM of human DP. These results suggest that P-LM-E8 fragments having the growth factor–binding capability of perlecan can recapitulate the composition of the BM surrounding DP and contribute to the integrated signal transduction from integrins and growth factor receptors expressed on DP. Considering the spatially regulated expression of LM isoforms in DP, the efficiency of induced differentiation on P-LM-E8 fragments, as well as the functionality of the induced cells, would depend on the selection of LM isoforms used as culture substrates.

In the present study, we compared the ability of iPSC-SKPs to differentiate into adipocytes, osteocytes, and Schwann cells as well as the gene expression signatures for SKPs with those induced on P-LM111-E8 fragment. Therefore, it needs to be confirmed whether the iPSC-SKPs induced on other P-LM-E8 fragments also possess the multipotency to differentiate into adipocytes, osteocytes, and Schwann cells and exhibit the gene expression signatures for SKPs. The iPSC-SKPs induced on P-LM111-E8 and other P-LM-E8 fragments are indistinguishable in their spindle-shaped morphology characteristic of SKPs and both equally express the established SKP marker proteins, nestin and fibronectin^18^. Therefore, it seems likely that the iPSC-SKPs induced on other P-LM-E8 fragments share the multipotency and the gene expression signatures for SKPs with those induced on P-LM111-E8 fragment, warranting further investigation.

The iPSC-SKPs differentiated on P-LM111-E8 fragment should also be compared with the SKPs isolated from human skin to consolidate their authenticity. Owing to the difficulty in using fresh human skin to isolate SKPs, we did not compare the iPSC-SKPs induced on P-LM111-E8 fragments with those from human skin. Furthermore, the SKP-induction efficiency on mouse feeder cells varies between experiments, depending on the condition of the iPSCs grown on mouse feeder cells (Supplementary Fig. S2). Therefore, it is difficult to obtain an adequate amount of iPSC-SKPs simultaneously with those on P-LM111-E8 fragment, hampering a direct comparison between the iPSC-SKPs induced on mouse feeder cells and those on P-LM-E8 fragments. The authenticity of the iPSC-SKPs induced on P-LM111-E8 and other P-LM-E8 fragments needs to be further addressed by direct comparison with SKPs from human skin to consolidate the advantage of the current protocol in clinical settings.

In conclusion, this study revealed that the efficiency of iPSC differentiation into SKPs is substantially improved by the modified LM-E8 fragments conjugated with growth factor-binding domain of perlecan. This finding offers a promising option for efficiently inducing differentiation into iPSC-SKPs for their clinical application, including the regeneration of human hair follicles.

## Materials and methods

### Human iPSC culture

Human iPS cell lines (201B7 and 1231A3) were obtained from the Center for iPS Cell Research and Application (Kyoto University). Human iPSCs were maintained on LM511-E8 (iMatrix-511; Nippi; 0.25 μg/cm^2^) in an uncoated manner, along with StemFit medium (Ajinomoto), in a humidified atmosphere of 5% CO_2_ and 95% air at 37 °C. At passage, the cells were dispersed in Accutase solution (BD Biosciences) for 5 min at 37 °C. Human iPSCs were seeded in a culture medium containing 10 μM Y-27632.

### LM-E8 and modified P-LM-E8 fragments

LM-E8 fragments were produced by transfecting FreeStyle 293-F cells with expression vectors encoding human LM α1-E8, α2-E8, α3-E8, α4-E8, α5-E8, β1-E8, β2-E8, β3-E8, γ1-E8, and γ2-E8 in defined combinations, as previously described^6,24,25^. An expression vector for a chimeric protein of LM α5-E8 and perlecan’s domain I (PDI) was constructed as previously described^22^. Expression vectors for chimeric proteins containing LMα1-E8, α2-E8, α3-E8, and α4-E8 were constructed by replacing the coding sequence for LMα5-E8 with those encoding LMα1-E8, α2-E8, α3-E8, and α4-E8. Recombinant LM-E8 and P-LM-E8 fragments were purified from the conditioned medium of transfected 293F cells using sequential chromatography on Ni–NTA-agarose (Qiagen) and anti-FLAG M2-agarose (Sigma) columns. The purified proteins were dialysed against phosphate-buffered saline (D-PBS; pH 7.4). Protein concentrations were determined with a BCA protein assay kit (Thermo Fisher Scientific), using bovine serum albumin as a standard.

### Generation of human iPSC-SKPs on LM-E8 and P-LM-E8 fragments

Before SKP differentiation, iPSCs were precultured on each LM-E8 fragment (0.5 µg/cm^2^) for 7 days. Coating of recombinant LM-E8 fragments was conducted by adding D-PBS to culture plates and then adding LM-E8 fragments at a density of 0.5 µg/cm^2^. The plates were then incubated at 37 °C for at least 1 h. When iPSC colonies reached 80%–90% confluence on each LM-E8 fragment, the cells were detached as described above and seeded at 1.4 × 10^2^ cells/cm^2^ in StemFit medium containing 10 µM Y-27632. After 24 h, the medium was switched to StemFit medium without liquid C, containing 500 ng/mL noggin (R&D Systems) and 10 µM SB431542 (Tocris Bioscience). After 5 days of culture, the StemFit medium was removed, and the cells were washed with D-PBS, after which they were cultured for 4 days in SKP medium containing DMEM/F12 (Thermo Fisher Scientific), 2% B27 supplement (Thermo Fisher Scientific), 100 units/mL penicillin and 100 µg/mL streptomycin (Thermo Fisher Scientific), 40 ng/mL bFGF (Wako), 20 ng/mL EGF (R&D Systems), and 3 µM CHIR99021 (Cayman Chemical). After 4 days of culture, the cells were dissociated using Accutase and subcultured on new uncoated dishes in SKP medium without CHIR99021.

The cell number was determined using Cell Counting Kit 8 (WST-8) (Abcam). iPSC-SKPs were added to a clear-bottom 96-well plate, and the number of cells was determined from the absorbance at 450 nm using a plate reader.

### RNA extraction and RT-PCR

The total RNA was extracted using RNeasy (Qiagen). cDNAs were synthesised by reverse transcription of 1 µg of total RNA using a high-capacity RNA-to-cDNA kit (Applied Biosystems). PCR was performed with KOD DNA polymerase (Toyobo) using the primers listed in Table 1 and the conditions described below. PCR amplification was performed as follows: 94 °C for 2 min, 25–35 cycles at 98 °C for 10 s, 60 °C or 63 °C for 30 s, and 68 °C for 30 s. The PCR products were separated on 1.5% agarose gels containing SYBR Safe DNA Gel Stain (Thermo Fisher Scientific). No specific amplification of the transcripts was detected from negative control reaction (without cDNA templates) using the PCR primers listed in Table 1 (see Supplementary Fig. S4).

**Table 1:** Primers used for RT-PCR.

Gene	Primers (forward/reverse; 5′–3′)
*Nestin*	cagcgttggaacagaggttg/gctggcacaggtgtctcaag
*Slug*	catctttggggcgagtgagtcc/cccgtgtgagttctaatgtgtc
*Sox9*	gtcagccaggtgctcaaagg/acttgtaatccgggtggtcc
*Dermo-1*	gcaagaagtcgagcgaagatg/ggcaatggcagcatcattcag
*BMP-4*	ttctgcagatgtttgggctgc/agagccgaagctctgcagag
*Wnt5a*	ggatggctggaagtgcaatg/acacaaactggtccacgatc
*Versican*	acgatgcctactttgccacc/tagtgaaacacaaccccatcc
*GAPDH*	cggagtcaacggatttggtcg/agccttctccatggtggtgaa

### Differentiation properties of iPSC-SKPs

For adipogenic and osteogenic differentiation, cells were seeded at 3 × 10^4^ cells/cm^2^ and allowed to adhere overnight in SKP medium. Adipogenic differentiation was induced for 2 weeks using a medium consisting of MEM (Thermo Fisher Scientific), 15% rabbit serum (Sigma Aldrich), 0.45 nM IBMX (Sigma Aldrich), 2.07 µM insulin (Sigma Aldrich), and 100 nM dexamethasone (Sigma Aldrich). All media were changed every 3–4 days. Cells were stained with Oil Red O solution (ScienCell Research Laboratories).

Osteogenic differentiation was induced for 2 weeks using a medium consisting of MEM (Thermo Fisher Scientific), 10% foetal bovine serum (Hyclone Laboratories), 100 nM dexamethasone (Sigma Aldrich), 10 mM β-glycerophosphate (Sigma Aldrich), and 50 µM l-ascorbic acid-2 phosphate (Sigma Aldrich). All media were changed every 3–4 days. Alkaline phosphatase (ALP) activity was determined using a Vector Blue Alkaline Phosphatase Substrate Kit (Vector Laboratories).

For Schwann cell differentiation, the cells were seeded at 0.5 × 10^4^ cells/cm^2^ on dishes coated with 0.02 mg/mL laminin (Sigma Aldrich) and 0.1 mg/mL poly-l-lysine (Sigma Aldrich) and allowed to adhere overnight in SKP medium. The cells were then cultured in a Schwann cell differentiation medium consisting of DMEM/F12 (Thermo Fisher Scientific), N2 supplement (Thermo Fisher Scientific), 5 µM forskolin (Sigma Aldrich), and 50 ng/mL heregulin-1β (Peprotech) for 3 weeks. All media were changed every 2–3 days. Immunofluorescence analysis was performed to examine the expression of S100β (Sigma Aldrich).

### Immunohistochemistry

The human studies were approved and conducted in accordance with policies of the Human Research Ethics Committee of Kao Corporation. Ethics approval number was #T005-170413. All procedures were carried out in accordance with the Declaration of Helsinki.

Anagen hair follicles were micro-dissected from normal occipital human scalp skin samples obtained from surgical operations with fully informed consent from the donors. The isolated HFs were immediately frozen in an OCT compound (Sakura Fine Technical) and cut to a thickness of 6 μm.

Human skin sections were fixed with 4% paraformaldehyde (Wako) for 15 min or acetone for 10 min and blocked with 10% goat serum (Nichirei Bioscience) in PBS for 1 h at room temperature (RT). Samples were incubated with primary antibodies for 2 h at RT before incubation with the appropriate secondary antibodies for 1 h at RT before counterstaining with haematoxylin (Sakura Fine Technical).

Immunofluorescence staining of the differentiated cells was performed as previously described^1^. In brief, cells were fixed with 4% paraformaldehyde (Wako) for 15 min, permeabilised with 0.1% Triton X-100 (Sigma Aldrich) for 5 min and blocked with 10% goat serum (Nichirei Bioscience) in PBS for 1 h at RT. The cells were incubated with primary antibodies for 2 h at RT before incubation with appropriate secondary antibodies for 1 h at RT, prior to counterstaining with Hoechst33342 (Dojindo Laboratories). The samples were observed under a Leica fluorescence microscope (Leica Microsystems). The antibodies used are listed in Table 2.

**Table 2:** Antibodies used for immunohistochemistry.

Antigen	Clone	Host species	Source	Dilution
LM α1	1F11	Mouse	Hattori et al.^26^	1:300
LM α 2	4H8-2	Rat	Abcam	1:100
LM α 3	2B10	Mouse	Fujiwara et al.^27^	1:300
LM α 4	2-11H	Mouse	Fujiwara et al.^28^	1:300
LM α 5	5D6	Mouse	Fujiwara et al.^27^	1:1000
LM β 1	LT3	Rat	Abcam	1:100
LM β 2	C4	Mouse	DSHB	1:500
LM β 3	#57	Mouse	Takashima et al.^29^	1:200
LM γ 1	A5	Rat	Abcam	1:100
LM γ 2	8C2	Mouse	Takashima et al.^29^	1:200
LM γ 3		Rabbit	Sigma Aldrich	1:50
Nestin	10C2	Mouse	Millipore	1:50
Fibronectin		Rabbit	Sigma Aldrich	1:50
αSMA	1A4	Mouse	Sigma Aldrich	1:50
S100β		Mouse	Sigma Aldrich	1:100
Histofine simple stain	MAX-PO (R)	Goat	Nichirei Bio science	Ready-to-use
Histofine simple stain	MAX-PO (MULTI)	Goat	Nichirei Bio science	Ready-to-use
Alexa Fluor 488 anti-mouse IgG		Goat	Thermo Fisher Scientific	1:200
Alexa Fluor 546 anti-rabbit IgG		Goat	Thermo Fisher Scientific	1:200

### Statistical analysis

Data are presented as mean ± standard error for each group. Statistical significance was analysed using Student’s *t*-test. All statistical analyses were conducted at a significance level of p < 0.001.

### Supplementary Information

Supplementary Figures.

## Data Availability

All data analysed during this study are included in this published article and the Supplementary Information file.

## Abbreviations

SKP: Skin-derived precursor cell
iPSC: induced pluripotent stem cell
DP: Dermal papilla
LM: Laminin
iPSC-SKP: iPSC-derived skin-derived precursor cell
ECM: Extracellular matrix
BM: Basement membrane
P-LM: Perlecan-conjugated laminin
ALP: Alkaline phosphatase
PBS: Phosphate-buffered saline
RT: Room temperature
IBMX: 3-Isobutyl-1-methylxanthine
PCR: Polymerase chain reaction
