# Supplementary material for: Laminin fragments conjugated with perlecan’s growth factor-binding domain differentiate human induced pluripotent stem cells into skin-derived precursor cells
Source: Sci Rep. 2023 Sep 4;13:14556. doi: 10.1038/s41598-023-41701-5 (PMC10477235; doi:10.1038/s41598-023-41701-5)
Supplement: Supplementary file 1 — Supplementary Figures. [file 41598_2023_41701_MOESM1_ESM.pdf]

Supplementary Information file

Laminin fragments conjugated with perlecan's growth factor–binding domain differentiate human induced pluripotent stem cells into skin-derived precursor cells

Yoriko Sugiyama-Nakagiri, Shiho Yamashita, Yukimasa Taniguchi, Chisei Shimono, Kiyotoshi Sekiguchi

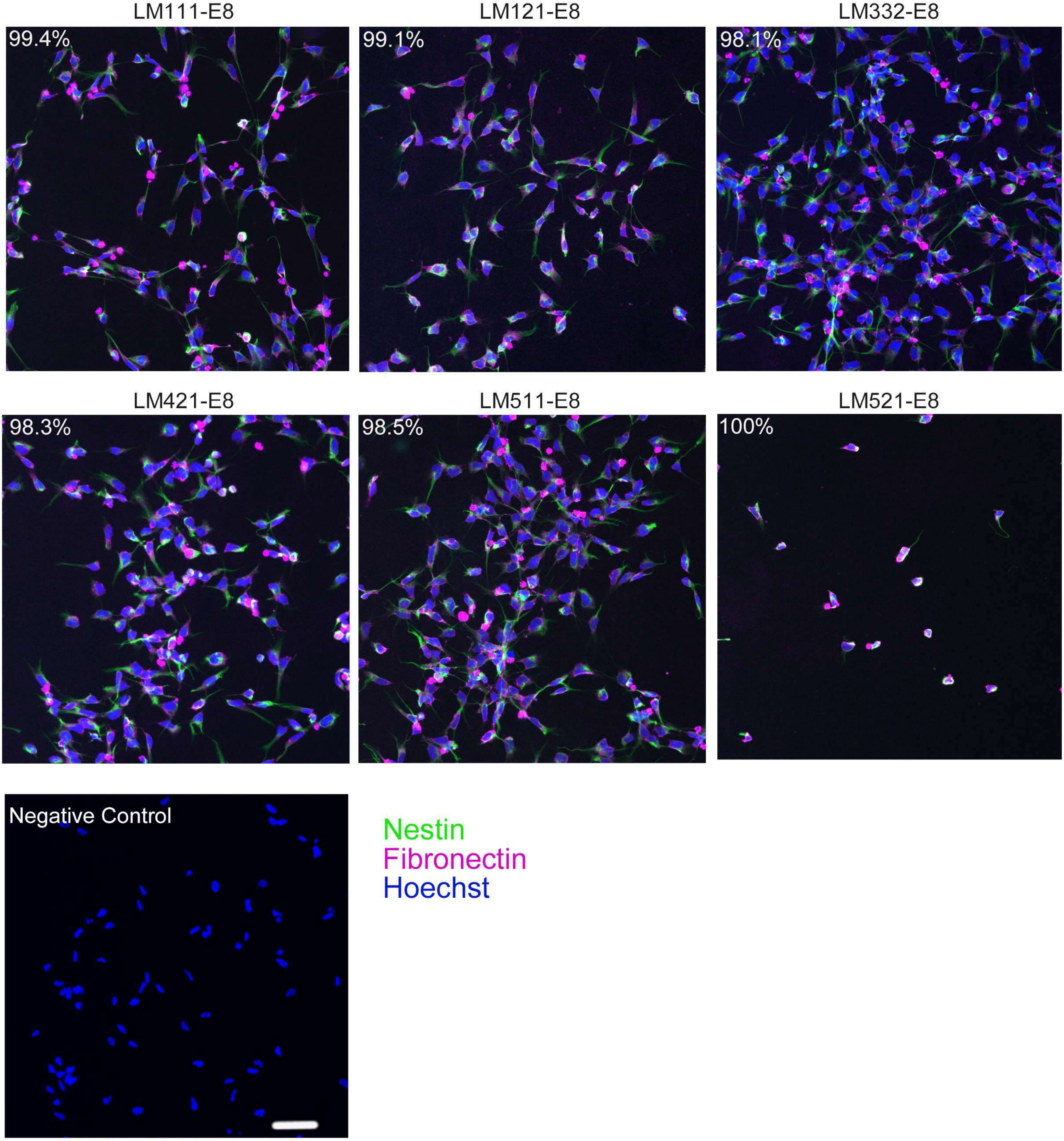

Figure S1. Magnified views of iPSC-SKPs (P1) immunostained for nestin and fibronectin.

The immunofluorescence images shown in Figure 2b are magnified to demonstrate that most, if not all, of the differentiated cells on various LM-E8 fragments were positive for nestin (green) and fibronectin (magenta), although staining for fibronectin was often less pronounced than that for nestin. The percentages of nestin-/fibronectin-positive cells are shown in the upper left corner of each photograph. Scale bar, 50 μm.

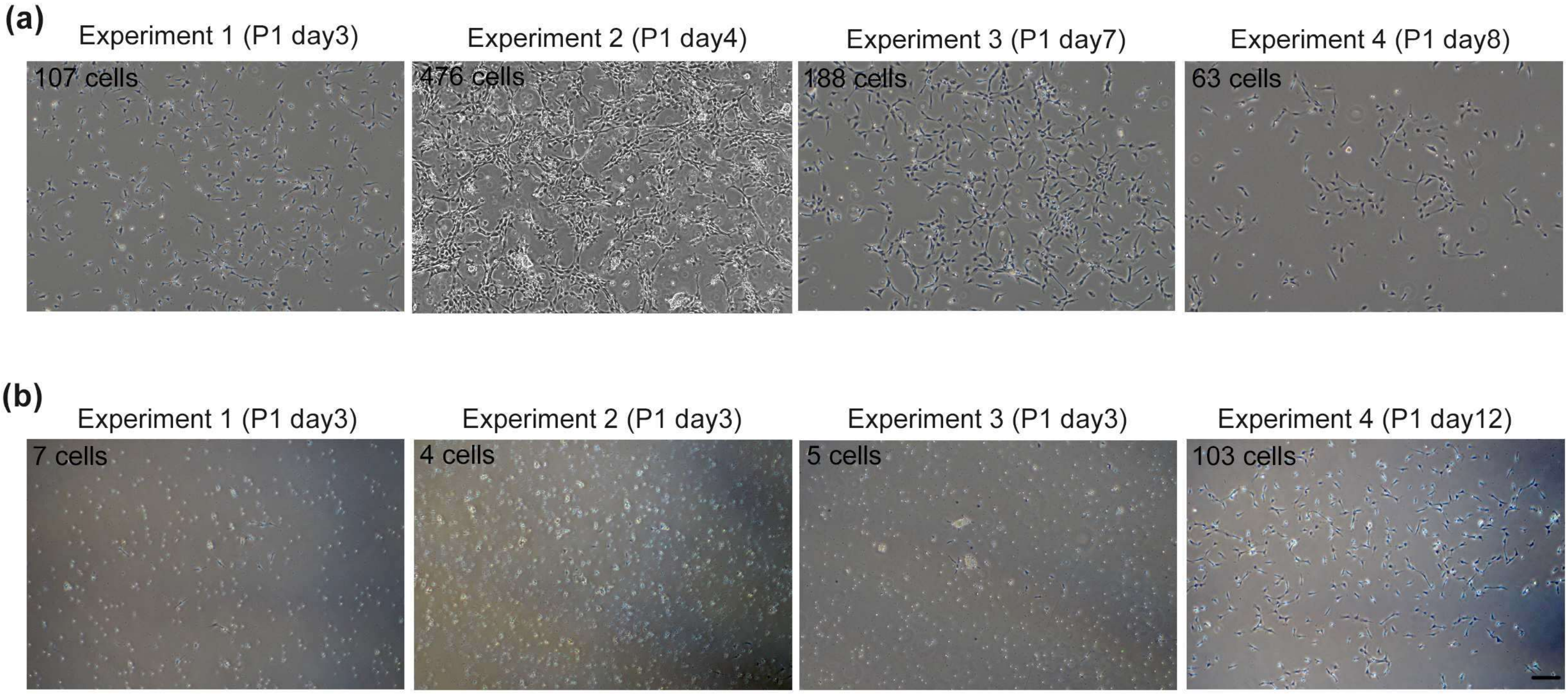

Figure S2. Differentiation induction efficiency on feeder cells and on LM511-E8 fragment. iPSCs (201B7) were induced to differentiate into iPSC-SKPs by the original protocol using mouse feeder cells (a) and the current protocol using LM511-E8 fragment (b). The induced cells on Day 9, that is, iPSC-SKPs (P0), were detached and replated on uncoated dishes, thereby selecting iPSC-SKPs. The resulting iPSC-SKPs (P1) were photographed on different days after replating as indicated. The differentiated cells showing spindle-shaped morphology were counted at three non-overlapping fields and expressed as the number of cells per square millimetre. The results of four independent experiments are presented for both protocols. The number of differentiated cells per square millimetre is indicated in the upper left corner of each photograph. Scale bar, 200 μm. The differentiation induction efficiency varies from experiment to experiment particularly on feeder cells, although the cells were photographed on different days after replating. Please note that iPSC-SKPs have a doubling time of approximately 24 h. The cells differentiated on LM511-E8 fragment were less adherent to the culture dishes and only a small fraction of the cells exhibited spindle-shaped morphology, an indicative of iPSC-SKPs. The number of spindle-shaped cells was apparently fewer on LM511-E8 fragment than on feeder cells, suggesting that differentiation induction efficiency was lower on LM511-E8 fragment.

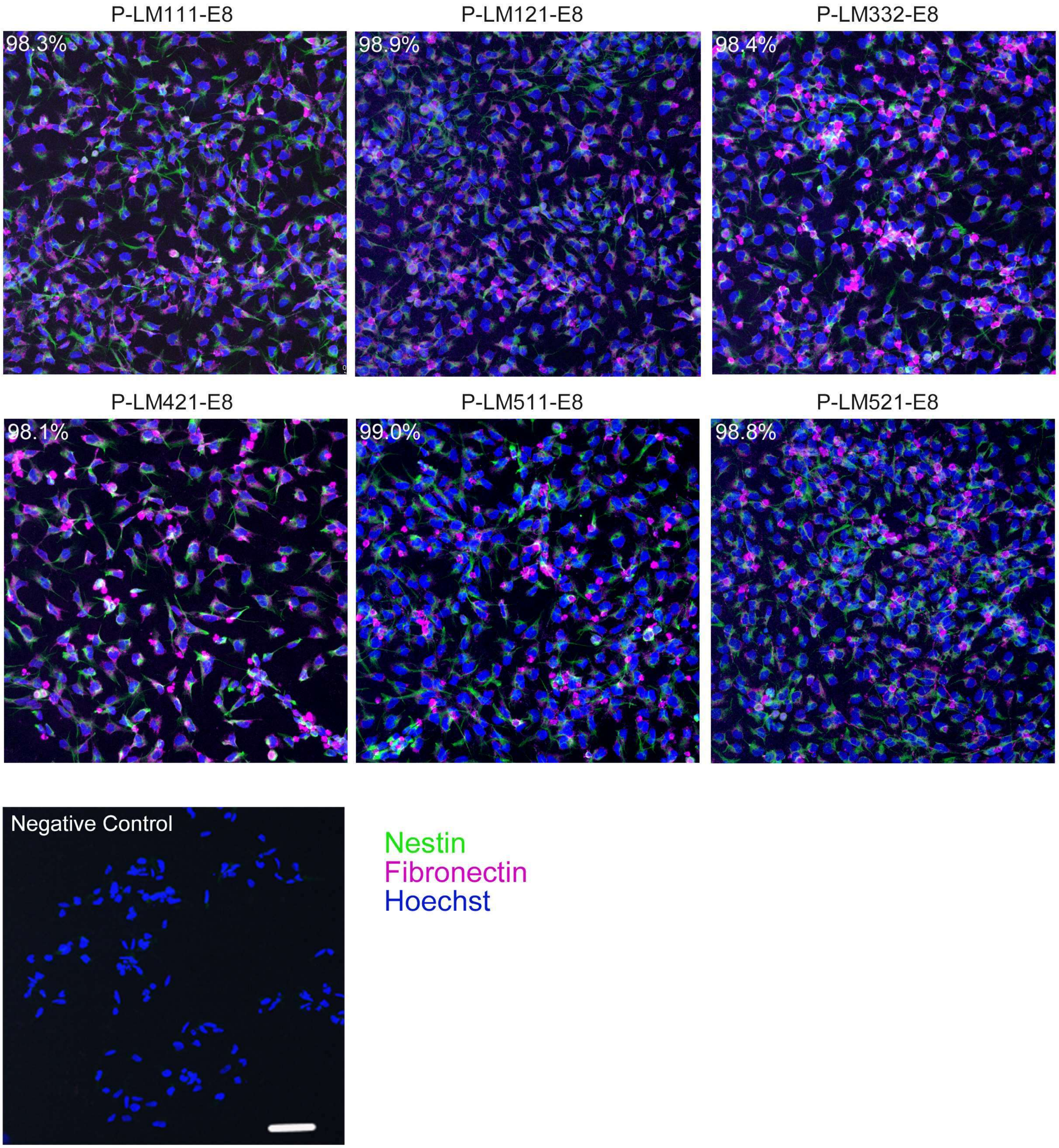

Figure S3. Magnified views of iPSC-SKPs (P1) immunostained for nestin and fibronectin.

The immunofluorescence images shown in Figure 3d are magnified to demonstrate that most of the differentiated cells on various P-LM-E8 fragments were positive for nestin (green) and fibronectin (magenta). The percentages of nestin-/fibronectin-positive cells are shown in the upper left corner of each photograph. Scale bar, 50 μm.

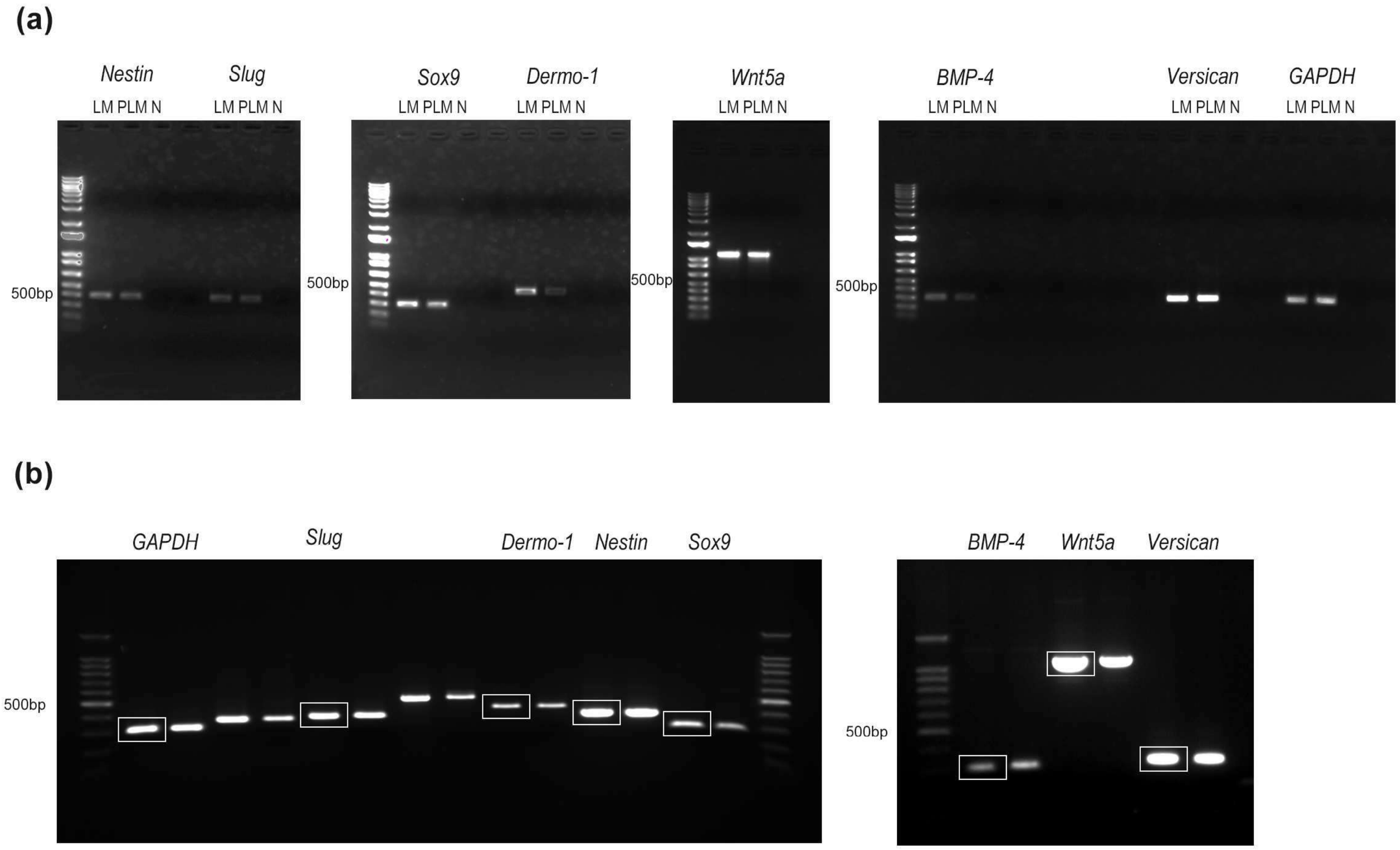

Figure S4. RT-PCR analyses of the iPSC-SKPs.

(a) The expression of the SKP marker genes in iPSC-SKPs differentiated on LM111-E8 (lanes LM) and P-LM111-E8 (lane PLM) fragments was detected using RT-PCR. Negative controls (i.e., no cDNA templates; lane N) were processed in parallel with other samples. Uncropped gel images are shown. No specific amplification of the transcripts was detected with negative controls.
(b) The expression of the same SKP marker genes were detected using RT-PCR in iPSC-SKPs differentiated on mouse feeder cells. The bands of interest are boxed in the uncropped gel images.

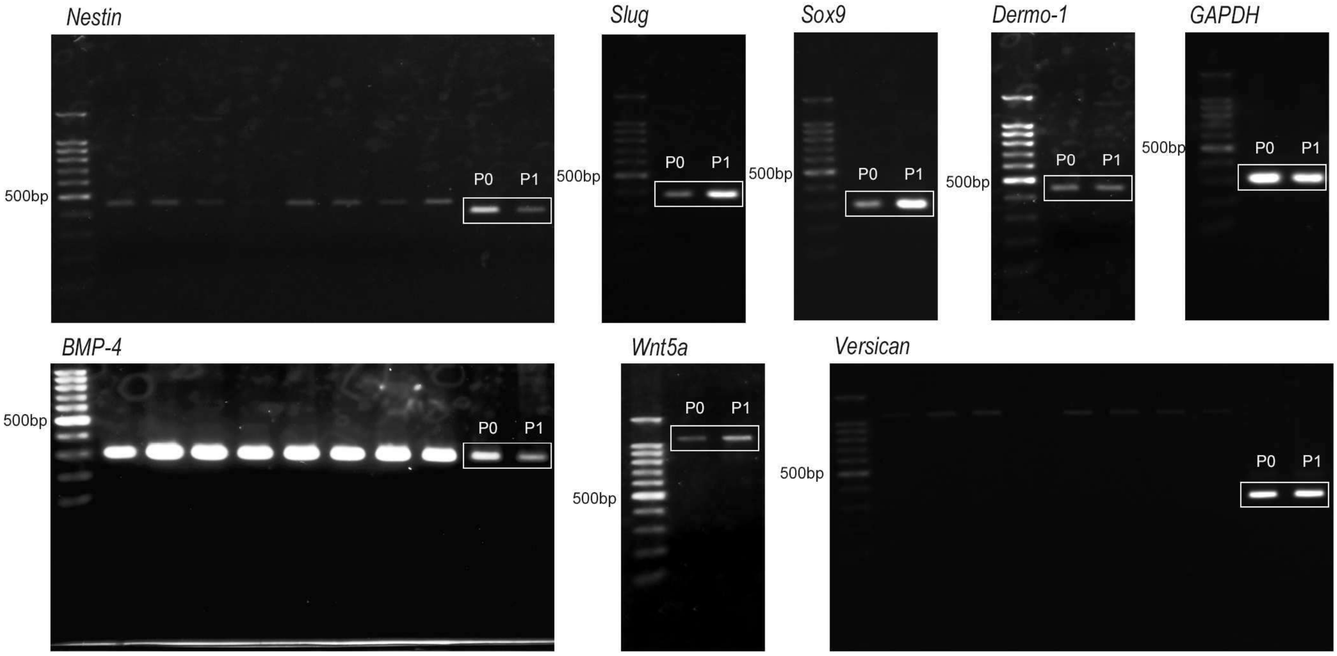

Figure S5. Uncropped gel images for Figure 4a.
The lanes shown in Fig. 4a are boxed.
